# Determinants of Ultra-Early Aspiration Pneumonia in Acute Intracerebral Hemorrhage Presenting to the Emergency Department

**DOI:** 10.3390/jcm15010226

**Published:** 2025-12-27

**Authors:** Giancarlo Ceccarelli, Luca Bortolani, Francesco Branda, Mattia Albanese, Maria Civita Cedrone, Francesco Baratta, Riccardo Renna, Giovanni Giordano, Anne Falcou, Antonio Sili Scavalli, Luigi Petramala, Gabriella d’Ettorre, Gioacchino Galardo

**Affiliations:** 1 Department of Public Health and Infectious Diseases, University of Rome Sapienza, and Azienda Ospedaliero Universitaria Policlinico Umberto I, 00161 Rome, Italy; giancarlo.ceccarelli@uniroma1.it (G.C.); luca.bortolani@uniroma1.it (L.B.); dott.albanese.mattia@gmail.com (M.A.); gabriella.dettorre@uniroma1.it (G.d.); 2 Medical Statistics and Molecular Epidemiology, University Campus Bio-Medico, 00100 Rome, Italy; f.branda@unicampus.it; 3 Emergency Medicine Unit, Department of Emergency-Acceptance, Critical Areas and Trauma, University of Rome Sapienza and Azienda Ospedaliero Universitaria Policlinico Umberto I, 00161 Rome, Italy; m.civitacedrone@policlinicoumberto1.it (M.C.C.); renna.1850997@studenti.uniroma1.it (R.R.); 4 Geriatric Unit, Department of Internal Medicine and Medical Specialties, Azienda Ospedaliero Universitaria Policlinico Umberto I, 00161 Rome, Italy; francesco.baratta@uniroma1.it; 5 Department of General and Specialistic Surgery, University of Rome Sapienza, 00161 Rome, Italy; giovanni.giordano@uniroma1.it; 6 Department of Medical and Cardiovascular Sciences, University of Rome Sapienza, 00161 Rome, Italy; a.falcou@policlinicoumberto1.it; 7 Stroke Unit, Emergency Department, Azienda Ospedaliero Universitaria Policlinico Umberto I, 00161 Rome, Italy; antonio.siliscavalli@uniroma1.it; 8 Department of Translational and Precision Medicine, University of Rome Sapienza, 00161 Rome, Italy

**Keywords:** intracerebral hemorrhage, aspiration pneumonia, stroke-associated pneumonia, emergency department, endotracheal intubation, neurological outcomes

## Abstract

**Background/Objectives**: Aspiration pneumonia is among the most frequent medical complications after intracerebral hemorrhage (ICH), yet its role during the ultra-early emergency department (ED) phase remains poorly understood. This study aimed to identify clinical and neurological factors independently associated with radiologically confirmed aspiration pneumonia in patients presenting with acute spontaneous ICH and to evaluate its association with early clinical outcomes. **Methods**: A retrospective observational cohort study was conducted in the neuro-emergency department of a large tertiary university hospital. All consecutive adults with spontaneous ICH confirmed by neuroimaging between January 2020 and December 2023 were included. Univariable and multivariable logistic regression models were used to identify independent predictors of pneumonia. **Results**: A total of 184 patients were analyzed (median age 74 years; 46% female). Radiologically confirmed aspiration pneumonia occurred in 37 patients (22.0%). Pneumonia was significantly associated with lower GCS, higher National Institutes of Health Stroke Scale (NIHSS) and ICH scores, shorter ED stay, and more frequent endotracheal intubation (ETI). In multivariable analysis, ETI (OR 5.42, 95% CI 1.57–18.63, *p* = 0.007), higher NIHSS score (OR 1.09, 95% CI 1.01–1.20, *p* = 0.047), and shorter ED stay (OR 0.97, 95% CI 0.95–0.99, *p* = 0.035) were independently associated with pneumonia. Aspiration pneumonia was not independently associated with neurosurgical referral (*p* = 0.082) or low GCS at discharge (*p* = 0.650). **Conclusions**: In this neuro-emergency cohort, aspiration pneumonia was common and strongly associated with neurological severity, particularly with endotracheal intubation and higher NIHSS scores. Although it did not independently predict early neurological deterioration or neurosurgical transfer, it identifies a critical period in which preventive measures—such as dysphagia screening, oral hygiene, and careful airway management—should be systematically applied. Larger multicenter studies with longer follow-up are needed to better define its long-term clinical consequences after ICH.

## 1. Introduction

Intracerebral hemorrhage (ICH) represents approximately 9–27% of all strokes and remains one of the most devastating forms of cerebrovascular disease, characterized by high early mortality and severe long-term disability [1,2]. In addition to primary brain injury, patients with ICH are particularly vulnerable to secondary systemic complications that significantly influence prognosis [2]. Among these, pneumonia (especially aspiration or aspiration pneumonia) is one of the most frequent and clinically relevant medical complications observed during the acute phase of stroke care [3].

Pneumonia following stroke, commonly referred to as stroke-associated pneumonia (SAP), arises from a complex interplay between neurological impairment, dysphagia, and neurogenic immunosuppression [4]. The incidence of SAP varies widely, ranging from 6% to 30% depending on diagnostic criteria and case mix [5]. In patients with ICH, the incidence appears even higher, reaching up to 25–30% in some cohorts and reflecting profound neurological depression, loss of protective airway reflexes, and an increased need for mechanical ventilation in this population [6,7].

The mechanisms linking acute brain injury and pulmonary infection are multifactorial. Depressed consciousness and loss of airway control facilitate aspiration of oropharyngeal or gastric contents, while stroke-induced immunodepression, mediated by sympathetic overactivity and hypothalamic–pituitary–adrenal axis activation, contributes to an increased susceptibility to bacterial infection [8]. Endotracheal intubation, frequently required in ICH for airway protection or for managing elevated intracranial pressure, further increases the risk through microaspiration and ventilator-associated pneumonia [8,9]. This complex pathophysiological network has been conceptualized as part of the “brain–lung crosstalk” phenomenon, where central nervous system injury precipitates systemic inflammatory and immune alterations that heighten pulmonary vulnerability [10].

Multiple prediction tools consistently highlight age, Glasgow Coma Scale (GCS) score, hematoma volume, and dysphagia as major drivers of pneumonia risk after ICH [11,12,13]. However, most of these models are derived from inpatient or intensive care populations and do not address the ultra-early phase of care within the Emergency Department (ED), where initial stabilization, airway management, and triage decisions occur. Evidence from this setting remains scarce, despite its critical role in determining both pneumonia risk and neurological outcome trajectories.

Furthermore, while stroke-associated pneumonia has been consistently linked to prolonged hospitalization, worse functional outcomes, and increased mortality [8], its independent impact during the immediate ED phase of ICH management is less clear. It remains uncertain whether aspiration pneumonia represents a direct causal factor of early deterioration or primarily a marker of severe neurological impairment.

To address these knowledge gaps, we conducted a retrospective observational study including all consecutive patients presenting with acute ICH to a tertiary neuro-emergency department between 2020 and 2023. Our objectives were to identify clinical and neurological factors independently associated with radiologically confirmed aspiration pneumonia and to assess its association with early outcomes, namely neurosurgical referral and neurological status at ED discharge. By focusing on this critical early phase, we aimed to clarify the interaction between neurological severity, airway management, and pneumonia in shaping the immediate trajectory of ICH care.

## 2. Materials and Methods

### 2.1. Design of the Study and Study Population

This cohort study included all consecutive adult patients (≥18 years) presenting with acute spontaneous ICH to the ED of a tertiary university hospital with a dedicated neuro-emergency unit between January 2020 and December 2023. Eligibility required radiological confirmation of spontaneous ICH on neuroimaging. Patients were excluded only if they had a history of respiratory symptoms in the days preceding or at the time of onset of acute neurological manifestations, if critical variables such as radiological findings, GCS scores, or outcome data were missing, or if they were transferred to another institution before completion of diagnostic and clinical assessments. Trained investigators using standardized electronic forms, ensuring completeness and consistency across cases, performed data collection.

For each patient, demographic information (age, sex, ethnicity) and clinical data at presentation were collected. Neurological severity on admission was assessed using the GCS, National Institutes of Health Stroke Scale (NIHSS), and ICH scores. Pre-admission events recorded within 24 h before arrival included transient loss of consciousness, vomiting, and epileptic seizure. Additional variables included the need for endotracheal intubation (ETI), the length of ED stay (measured in hours from arrival to discharge or transfer), the destination unit (neurosurgical versus other wards), and in-ED mortality. The GCS score at ED discharge was used as an indicator of early neurological outcome.

Radiologically confirmed aspiration pneumonia was defined as the presence of new pulmonary infiltrates on chest imaging consistent with aspiration, accompanied by compatible clinical signs such as fever, cough, or hypoxia, as evaluated by the treating physician and confirmed by radiology review, occurring within the first 72 h after ED admission and absent at presentation [14].

The primary objective of the study was to identify clinical and neurological variables independently associated with aspiration pneumonia in patients with ICH. Secondary objectives included evaluating whether pneumonia influenced the likelihood of transfer to the neurosurgical unit or was associated with neurological deterioration at ED discharge, defined as a GCS score below 13. The threshold of <13 was chosen based on the cohort median to allow for dichotomization and adequate statistical power.

The study design followed the ethical standards of the institutional research committee and adhered to the principles of the Declaration of Helsinki. Reporting was performed in accordance with the STROBE guidelines for observational studies. The institutional ethics committee (Prot 0058/2025) of the hospital approved the study protocol. All data was handled anonymously to ensure confidentiality and compliance with data protection standards.

### 2.2. Statistical Analysis

Descriptive statistics were used to summarize participants’ demographic and clinical characteristics. Categorical variables are presented as frequencies and percentages, while continuous variables are expressed as medians with interquartile ranges (IQR). Group differences were assessed using Pearson’s chi-squared test for categorical variables and the Mann–Whitney U test for non-normally distributed continuous variables. Multivariable logistic regression analyses were performed to identify factors independently associated with pneumonia and to evaluate their impact on clinical outcomes.

Three models were built: in the first, the dependent variable was the diagnosis of aspiration pneumonia; in the second, transfer to the neurosurgical unit; and in the third, a low GCS score at ED discharge. To increase statistical power, a cut-off at the median value (<13) was applied to define low GCS at discharge as a dichotomous variable. Independent variables were included in the multivariable models if considered clinically relevant or if they showed a *p*-value < 0.10 in univariate analyses. Some variables that were significant at univariate analysis were not included in the multivariable models due to collinearity with other indicators of clinical severity; in such cases, clinically more representative variables were retained to avoid redundancy and improve model stability. Considering the limited number of aspiration pneumonia events, multivariable analyses were intentionally restricted to a small set of clinically meaningful covariates to reduce the risk of overfitting. Variable selection was therefore conservatively guided by clinical relevance and effect size rather than univariate statistical significance alone. Results are reported as odds ratios (OR) with 95% confidence intervals (CI).

A two-sided *p*-value < 0.05 was considered statistically significant. All analyses were conducted using Stata/SE version 19.5 (StataCorp LLC, College Station, TX, USA).

## 3. Results

### 3.1. Characteristics of the Study Population

A total of 184 participants were included in the analysis. Detailed demographic and clinical characteristics are shown in Table 1.

Within the 24 h preceding admission, 60 individuals (32.6%) reported a transient loss of consciousness, 58 (31.5%) reported vomiting, and 14 (7.6%) experienced an epileptic seizure.

At ED presentation, median scores were 13 (IQR 9–15) for the GCS, 2 (IQR 1–3) for the ICH score, and 14 (IQR 6–22) for NIHSS. The median ED length of stay was 23 h (IQR 8.3–40.8).

Overall, 27 patients (14.7%) were transferred to the neurosurgical unit, while the remainder were admitted to other wards and the median GCS score at ED discharge was 13 (IQR 5–15). Twenty-nine participants (15.8%) died in the ED. Endotracheal intubation was performed in 32 participants (17.4%).

A radiological diagnosis of aspiration pneumonia, not present at emergency department (ED) admission but developing within the first 72 h after the acute neurological event, was confirmed in 37 participants (22.0%). Pneumonia status was unavailable for 16 participants. When performed, endotracheal intubation was undertaken for airway protection in the context of neurological deterioration and was not a consequence of pneumonia.

### 3.2. Associations Between Aspiration Pneumonia and Clinical Characteristics

Comparative analyses between patients with and without aspiration pneumonia are summarized in Table 2. The occurrence of pneumonia was not significantly associated with sex (*p* = 0.651) or age (*p* = 0.705). A borderline association was observed with seizures at ED presentation (*p* = 0.056). Neither transient loss of consciousness (*p* = 0.292) nor vomiting (*p* = 0.183) alone were significantly associated with pneumonia; however, when the two were combined into a single variable, the association approached significance (*p* = 0.060).

Patients with pneumonia had significantly lower GCS scores at ED admission (median 11 vs. 13, *p* = 0.035), but not at ED discharge (*p* = 0.331). Pneumonia was also associated with higher neurological severity, as reflected by higher ICH (median 2 vs. 1.5, *p* = 0.006) and NIHSS scores (median 20 vs. 13, *p* = 0.006). Moreover, these patients had a larger hemorrhage volume (median 30 vs. 17.8 mL, *p* = 0.013), a shorter ED stay (median 13.5 vs. 25.6 h, *p* = 0.001) and were significantly more likely to require endotracheal intubation (40.5% vs. 9.9%, *p* < 0.001).

No significant difference was observed in in-ED mortality between patients with and without pneumonia (13.5% vs. 16.0%, *p* = 0.709). However, neurosurgical referral was significantly more frequent among those with pneumonia (29.7% vs. 9.9%, *p* = 0.003). All associations are reported in detail in Table 2.

### 3.3. Multivariable Analyses

Three multivariable logistic regression models were constructed to address distinct but complementary clinical questions regarding aspiration pneumonia and its implications in acute neurological emergencies.

The first model explored factors independently associated with the development of aspiration pneumonia, serving as a proxy for the pathophysiological and procedural risk of aspiration during the acute phase. Variables included markers of neurological severity, airway protection, and early management dynamics.

The second model investigated predictors of transfer to the neurosurgical unit, using this outcome as a proxy for clinical decision-making and the impact of respiratory complications on referral pathways. This model assessed whether the occurrence of aspiration pneumonia influenced triage and treatment allocation decisions.

The third model examined predictors of low GCS score at ED discharge, considered a proxy for early neurological outcome and for the potential short-term neurological consequences of aspiration and respiratory compromise.

Together, these models provided a multidimensional assessment of aspiration pneumonia, from its determinants to its clinical and functional implications. Comprehensive regression results, including all variables and effect estimates, are provided in Table 3.

#### 3.3.1. Model 1—Predictors of Aspiration Pneumonia

In the first model, ETI (odds ratio [OR] 5.42, 95% confidence interval [CI] 1.57–18.63, *p* = 0.007), ED length of stay (OR 0.97, 95% CI 0.95–0.99, *p* = 0.035), and NIHSS score (OR 1.09, 95% CI 1.01–1.20, *p* = 0.047) were independently associated with pneumonia. Age, sex, GCS at admission, and the presence of transient loss of consciousness and/or vomiting were not significant predictors.

#### 3.3.2. Model 2—Predictors of Transfer to the Neurosurgical Unit

In the second model, aspiration pneumonia was not significantly associated with neurosurgical transfer (*p* = 0.082). Instead, older age (OR 0.93 per year, 95% CI 0.90–0.97, *p* = 0.001) and longer ED stay (OR 0.94 per hour, 95% CI 0.90–0.99, *p* = 0.015) were independently and inversely associated with neurosurgical referral. Sex and GCS at admission were not significant predictors.

#### 3.3.3. Model 3—Predictors of Low GCS at ED Discharge

In the third model, a low GCS score at ED discharge (<13) was independently associated with older age (OR 1.05 per year, 95% CI 1.02–1.09, *p* = 0.001) and lower baseline GCS at admission (OR 0.79 per point, 95% CI 0.72–0.88, *p* < 0.001). Aspiration pneumonia was not associated with neurological deterioration (*p* = 0.650), nor were sex or ED length of stay.

## 4. Discussion

In this observational cohort of 184 older adults presenting with acute neurological insult to the emergency department, we found that radiologically confirmed aspiration pneumonia occurred in 22.0% of patients. This incidence falls within the range reported in prior stroke-associated pneumonia (SAP) series (11–31%) and is consistent with earlier reports of pneumonia as one of the most frequent post-stroke complications [15,16]. The clustering of pneumonia among patients with greater neurological severity underscores the tight coupling between brain injury, airway compromise, and early pulmonary complications in neuro-emergency settings.

### 4.1. Determinants of Aspiration Pneumonia

On univariable analyses, pneumonia was more frequent in patients with lower GCS at admission, higher NIHSS and ICH scores, and a higher frequency of endotracheal intubation (ETI). In multivariable modeling, three factors remained independently associated with pneumonia: ETI, higher NIHSS, and shorter ED length of stay.

The strong association of ETI with pneumonia is biologically plausible. Intubation bypasses physiological airway defenses and can predispose to microaspiration, particularly in patients with depressed airway reflexes or gastric regurgitation. Moreover, ventilator-associated pneumonia (VAP) is a well-described complication in neurocritical and stroke populations, with incidences reported between 20% and 60% depending on setting and duration of mechanical ventilation [17]. Thus, ETI may reflect both a marker of severe neurological impairment and a procedural risk factor for pulmonary complications.

The independent association of the NIHSS score lends further support to a severity-driven mechanism: larger strokes or hemorrhages are more likely to impair level of consciousness, swallowing, and cough reflex, thereby facilitating aspiration. Prior studies have consistently identified stroke severity (often operationalized by NIHSS) as a strong predictor of SAP [5,18,19,20]. The inverse association between ED length of stay and pneumonia is somewhat paradoxical at first glance, but is likely explained by process-of-care dynamics: patients with more severe presentations who require urgent imaging, airway management, or intensive care transfer are managed expediently, resulting in shorter ED exposure windows. In other words, rapid triage and disposition may mediate the observed “protective” association, rather than a true causal effect of shorter observation time.

Interestingly, baseline GCS on admission lost significance in the fully adjusted model (*p* = 0.063) despite being significantly lower in pneumonia cases on univariable comparison. This attenuation is likely due to collinearity and shared explanatory variance among GCS, NIHSS, and ETI, which capture overlapping aspects of neurological compromise. In practice, including highly correlated severity metrics in the same model can suppress individual associations. A more parsimonious or dimension-reduced modeling approach may mitigate this issue.

### 4.2. Pneumonia and Immediate Neurological Outcomes

When neurosurgical transfer was the outcome, pneumonia did not remain significantly predictive (*p* = 0.082). Instead, age (OR 0.93 per year, *p* = 0.001) and longer ED stay (OR 0.94 per hour, *p* = 0.015) were inversely associated with neurosurgical referral. This suggests that surgical candidacy decisions may be influenced more by age, comorbidities, or logistical considerations rather than the presence of early pneumonia.

Similarly, in the model of low GCS at ED discharge (<13), pneumonia showed no independent association (OR 0.81, *p* = 0.650). In contrast, baseline GCS (OR 0.79 per point, *p* < 0.001) and age (OR 1.05 per year, *p* = 0.001) remained significant. The lack of association between pneumonia and immediate neurological status is understandable in light of temporal constraints: pneumonia likely exerts its deleterious effects over a longer timeframe (e.g., via systemic inflammation, hypoxia, metabolic stress) rather than manifesting within the few hours to a day of ED observation. Moreover, competing events (death, sedation, transfer) may attenuate associations measurable at discharge.

Nonetheless, the absence of association with short-term neurological outcome should not be taken to imply that pneumonia is clinically inconsequential. In the broader literature, SAP is consistently linked with worse in-hospital mortality, longer hospitalization, and poorer functional recovery [21,22]. Our findings likely reflect the limited window and competing risks inherent to ED-based observational endpoints.

### 4.3. Potential Clinical Implications

From a clinical perspective, our findings suggest potential implications for the early management of patients with acute ICH in the emergency department. The observed associations between aspiration pneumonia and markers of neurological severity, together with the frequent need for airway protection, indicate that this ultra-early phase of care may represent an opportunity for preventive interventions. While causal relationships cannot be inferred, measures such as early attention to swallowing function when feasible, aspiration precautions, oral hygiene, patient positioning, and careful airway management could be considered in patients identified as being at higher risk. In this context, aspiration pneumonia may serve as a clinical signal of vulnerability, helping to inform risk stratification and supportive care decisions.

Importantly, the clinical relevance of aspiration pneumonia is unlikely to be confined to the emergency department phase alone. Its potential impact on longer-term neurological and functional outcomes cannot be excluded. Pneumonia may contribute to delayed neurological recovery through mechanisms such as systemic inflammation, hypoxia, metabolic stress, and prolonged immobilization, which typically manifest beyond the ultra-early emergency department phase. Future prospective and longitudinal studies with extended follow-up are therefore needed to evaluate the relationship between aspiration pneumonia and longer-term neurological outcomes, including functional status, disability, and mortality after intracerebral hemorrhage.

### 4.4. Methodological Strengths, Limitations and Potential Confounders

This study has several strengths, including its focus on the early risk of aspiration pneumonia, the availability of a well-defined neuro-emergency cohort, and the use of multivariable modeling to account for potential confounding factors. Nevertheless, some limitations should be acknowledged when interpreting these findings.

First, the relatively small number of pneumonia events (n = 37) limits the events-per-variable ratio and may have led to model overfitting and unstable coefficient estimates. The wide confidence intervals, particularly for ETI, reflect this uncertainty. In future analyses, penalized regression approaches, such as Firth’s correction, could enhance the robustness and reliability of estimates.

Second, the potential collinearity among severity indicators, including GCS, NIHSS, and ETI, which are inherently interrelated measures of neurological impairment. Such multicollinearity can generate suppression effects or unstable estimates, thereby obscuring the true magnitude of associations. Future models should therefore assess variance inflation factors and prioritize the inclusion of non-overlapping predictors to improve model stability.

Third, although for the entire cohort the interval between symptom onset and emergency department evaluation was less than 24 h, this timing could not be reliably reconstructed for all patients. While in many cases presentation occurred within a few hours, in others the information was unavailable or could not be estimated with sufficient accuracy. To avoid potential misclassification bias, this variable was therefore not included in the analyses.

Another limitation of this study is the lack of microbiological data and the absence of a focused analysis on antibiotic treatments. However, it should be noted that the present investigation was specifically designed to capture the very early phase following the acute neurological event, a time window in which therapeutic interventions are typically limited to empirical antibiotic coverage and microbiological results are not yet available. From a microbiological standpoint, it is also important to acknowledge that identifying a causative pathogen in suspected aspiration pneumonia is inherently challenging. Patients often fail to produce adequate sputum samples and are frequently too weak to cough effectively, while invasive procedures such as bronchoscopy or bronchoalveolar lavage (BAL) are contraindicated or impractical in this population. Consequently, microbiological confirmation is rarely achieved, even in clinically and radiologically consistent cases. Nevertheless, future prospective studies should incorporate systematic microbiological testing whenever feasible, in order to better characterize the etiology of aspiration pneumonia and to inform targeted antimicrobial strategies.

A further potential source of bias relates to imaging: in asymptomatic patients, or in those without clinical, laboratory, or radiological evidence of pneumonia, chest computed tomography (CT) was not routinely performed, which may have led to underestimation of subclinical or radiographically silent aspiration events.

Finally, beyond the direct aspiration hypothesis, emerging evidence supports a brain–lung crosstalk paradigm in acute brain injury, in which neurological damage triggers systemic inflammatory responses, autonomic dysregulation, and pulmonary endothelial alterations that increase lung vulnerability [10,23]. While the present study did not directly explore this mechanism, the neurogenic component of pulmonary injury should be regarded as a potential confounder in the interpretation of the observed associations. Neuroinflammatory and autonomic pathways activated by acute brain injury could mimic or potentiate the effects attributed to aspiration, thereby overestimating the apparent causal weight of aspiration itself. According to the “double hit” model, the primary brain insult primes the lung for injury, and a subsequent event such as aspiration or mechanical ventilation precipitates overt pulmonary dysfunction [10,23]. This bidirectional brain–lung interaction highlights that pneumonia in neurologically compromised patients may not merely represent a bystander phenomenon but rather a pathophysiological amplifier of systemic and neurological stress.

Future prospective and longitudinal studies are warranted to strengthen causal inference and to capture longer-term clinical outcomes beyond the emergency department phase, which could not be addressed within the retrospective and ultra-early scope of the present study.

## 5. Conclusions

This study examines a very early and rarely explored phase of care, the ED. In this acute neuro-emergency context, aspiration pneumonia appears to function less as a direct cause of early deterioration and more as a clinical marker of underlying neurological severity, with the observed associations reflecting early management dynamics rather than direct cause–effect relationships. Nevertheless, its occurrence delineates a critical window of vulnerability during which prompt aspiration prevention and meticulous airway management may significantly influence the patient’s subsequent clinical course.

Looking ahead, future research should extend observation beyond the emergency setting to include inpatient and post-discharge outcomes, such as functional recovery, in-hospital mortality, and respiratory sequelae. Larger multicenter cohorts would enable stratified analyses by stroke subtype, lesion location, and ventilatory exposure. From a methodological perspective, future studies will also incorporate alternative modeling suited for limited event counts, to further validate and refine the observed associations. Ultimately, pneumonia may exert clinically meaningful downstream effects that warrant prospective evaluation and targeted preventive strategies in neuro-emergency care.

## Figures and Tables

**Table 1:** Characteristics of enrolled participants.

	n = 184	(%) [IQR]
Age		
Years (median [IQR])	77	[67.5–84.5]
Gender		
Male	103	(56.0)
Female	81	(44.0)
Race		
Caucasian	171	(92.9)
Others *	13	(7.1)
Radiological diagnosis of ab ingestis pneumonia		
Yes	37	(22.0)
GCS score at admission		
(median [IQR])	13	[9–15]
Transient loss of consciousness		
Yes	60	(32.2)
Vomiting		
Yes	58	(31.5)
Epileptic seizure		
Yes	14	(7.6)
Length of stay in the ED		
Hours (median [IQR])	23	[8.3–40.8]
ETI		
Yes	32	(17.4)
ICH score		
(median [IQR])	2	[1–3]
NIHSS score		
(median [IQR])	14	[6–22]
Hemorrhage location		
Lobar	121	(65.8)
Deep	47	(25.5)
Cerebellar	11	(6.0)
Brainstem	5	(2.7)
Hemorrhage laterality		
Right	84	(45.7)
Left	84	(45.7)
Bilateral	16	(8.6)
Hemorrhage volume		
mL (median [IQR])	21.6	[6.8–46.8]
Died in the ED		
Yes	29	(15.8)
GCS score at discharge		
(median [IQR])	13	[5–15]
Unit of destination		
Neurosurgical unit	27	(14.7)
Other units	128	(69.6)

* “Others” category includes East, South and Southeast Asian; African, Caribbean and North American black; and Latin American. Note: data on pneumonia were missing for 16 patients; percentages were calculated on available cases, and totals may not sum to the overall study population. Abbreviations: n, number; IQR, interquartile range; GCS, Glasgow Coma Scale; ED, emergency department; ETI, endotracheal intubation; ICH, intracerebral hemorrhage; NIHSS, National Institutes of Health Stroke Scale.

**Table 2:** Associations between pneumonia and participants characteristics.

	Pneumonia (n = 37)		No Pneumonia (n = 131)		
	n	(%)	n	(%)	*p*-Value
Age					
Years (median [IQR])	78	[69–82]	77	[67–85]	0.705
Gender					
Male	23	(62.2)	76	(58.0)	
Female	14	(37.8)	55	(42.0)	0.651
GCS score at admission					
(median [IQR])	11	[6–14]	13	[10–15]	0.035 *
Transient loss of consciousness					
Yes	15	(40.5)	41	(31.3)	
No	22	(59.5)	90	(68.7)	0.292
Vomiting					
Yes	15	(40.5)	38	(29.0)	
No	22	(59.5)	93	(71.0)	0.183
Transient loss of consciousness and/or vomiting					
Yes	24	(64.9)	62	(47.3)	
No	13	(35.1)	69	(52.7)	0.060
Epileptic seizure					
Yes	0	(0.0)	12	(9.2)	
No	37	(100.0)	119	(90.8)	0.056
Length of stay in the ED					
Hours (median [IQR])	13.5	[4.3–26.1]	25.6	[10.0–46.7]	0.001 *
ETI					
Yes	15	(40.5)	13	(9.9)	
No	22	(59.5)	118	(90.1)	<0.001 *
ICH score					
(median [IQR])	2	[2,3]	1.5	[0–3]	0.006 *
NIHSS score					
(median [IQR])	20	[14–28]	13	[5–21]	0.006 *
Hemorrhage location					
Lobar	20	(54.1)	90	(68.7)	
Deep	12	(32.4)	31	(23.7)	
Cerebellar	4	(10.8)	6	(4.6)	
Brainstem	1	(2.7)	4	(3.1)	0.295
Hemorrhage laterality					
Right	17	(45.9)	61	(46.6)	
Left	16	(43.2)	59	(45.0)	
Bilateral	4	(10.9)	11	(8.4)	0.900
Hemorrhage volume					
mL (median [IQR])	30	[18.5–51.3]	17.8	[4.8–42.7]	0.013 *
Died in the ED					
Yes	5	(13.5)	21	(16.0)	
No	32	(86.5)	110	(84.0)	0.709
GCS score at discharge					
(median [IQR])	10	[5–14]	13	[5–15]	0.331
Unit of destination					
Neurosurgical unit	11	(29.7)	13	(9.9)	
Other units	21	(56.8)	97	(74.0)	0.003 *

* Statistically significant. Note: data on pneumonia were missing for 16 patients; percentages were calculated on available cases, and totals may not sum to the overall study population. Abbreviations: n, number; IQR, interquartile range; GCS, Glasgow Coma Scale; ED, emergency department; ETI, endotracheal intubation; ICH, intracerebral hemorrhage; NIHSS, National Institutes of Health Stroke Scale.

**Table 3:** Multivariable logistic regression models assessing factors associated with aspiration pneumonia, transfer to neurosurgical unit and low GCS at discharge (<13).

	Aspiration Pneumonia			Transfer to Neurosurgical Unit			Low GCS at Discharge (<13)		
	OR	(95% C)	*p*-Value	OR	(95% CI)	*p*-Value	OR	(95% CI)	*p*-Value
Age	1.02	(0.99–1.06)	0.203	0.93	(0.90–0.97)	0.001 *	1.05	(1.02–1.09)	0.001 *
Gender (ref: male)	0.97	(0.41–2.32)	0.949	0.94	(0.29–3.09)	0.925	1.01	(0.48–2.14)	0.976
GCS score at admission	1.20	(0.99–1.45)	0.063	0.91	(0.78–1.06)	0.211	0.79	(0.72–0.88)	<0.001 *
Transient loss of consciousness and/or vomiting	1.06	(0.39–2.91)	0.911	-	-	-	-	-	-
Length of stay in the ED	0.97	(0.95–0.99)	0.035 *	0.94	(0.90–0.99)	0.015 *	1.01	(0.99–1.02)	0.369
ETI	5.42	(1.57–18.63)	0.007 *	-	-	-	-	-	-
NIHSS score	1.09	(1.01–1.20)	0.047 *	-	-	-	-	-	-
Aspiration pneumonia	-	-	-	2.95	(0.87–10.00)	0.082	0.81	(0.33–2.01)	0.650

* Statistically significant. Abbreviations: OR, odds ratio; CI, confidence interval; GCS, Glasgow Coma Scale; ED, emergency department; ETI, endotracheal intubation; NIHSS, National Institutes of Health Stroke Scale.

## Data Availability

All data are comprehensively presented in the Section 3; nevertheless, they can be made available by the corresponding author upon reasonable and justified request.

## Abbreviations

The following abbreviations are used in this manuscript:

BAL	Bronchoalveolar Lavage
CI	Confidence Interval
CT	Computed Tomography
ED	Emergency Department
ETI	Endotracheal Intubation
GCS	Glasgow Coma Scale
ICH	Intracerebral Hemorrhage
IQR	Interquartile Range
IRB	Institutional Review Board
NIHSS	National Institutes of Health Stroke Scale
OR	Odds Ratio
SAP	Stroke-Associated Pneumonia
SIDS	Stroke-Induced Immunodepression Syndrome
VAP	Ventilator-Associated Pneumonia
